# Doxorubicin increases the effectiveness of Apo2L/TRAIL for tumor growth inhibition of prostate cancer xenografts

**DOI:** 10.1186/1471-2407-5-2

**Published:** 2005-01-07

**Authors:** Ahmed El-Zawahry, John McKillop, Christina Voelkel-Johnson

**Affiliations:** 1 Department of Microbiology & Immunology, Medical University of South Carolina, Charleston SC, USA

## Abstract

**Background:**

Prostate cancer is a significant health problem among American men. Treatment strategies for androgen-independent cancer are currently not available. Tumor necrosis factor-related apoptosis-inducing ligand (Apo2L/TRAIL) is a death receptor ligand that can induce apoptosis in a variety of cancer cell lines, including androgen-independent PC3 prostate carcinoma cells. *In vitro*, TRAIL-mediated apoptosis of prostate cancer cell lines can be enhanced by doxorubicin and correlates with the downregulation of the anti-apoptotic protein c-FLIP. This study evaluated the effects of doxorubicin on c-FLIP expression and tumor growth in combination with Apo2L/TRAIL in a xenograft model.

**Methods:**

*In vitro *cytotoxic effects of TRAIL were measured using a MTS-based viability assay. For *in vivo *studies, PC3 prostate carcinoma cells were grown subcutaneously in athymic nude mice and tumor growth was measured following treatment with doxorubicin and/or Apo2L/TRAIL. c-FLIP expression was determined by western blot analysis. Apoptosis in xenografts was detected using TUNEL. Statistical analysis was performed using the student t-test.

**Results:**

*In vitro *experiments show that PC3 cells are partially susceptible to Apo2L/TRAIL and that susceptibility is enhanced by doxorubicin. In mice, doxorubicin did not significantly affect the growth of PC3 xenografts but reduced c-FLIP expression in tumors. Expression of c-FLIP in mouse heart was decreased only at the high doxorubicin concentration (8 mg/kg). Combination of doxorubicin with Apo2L/TRAIL resulted in more apoptotic cell death and tumor growth inhibition than Apo2L/TRAIL alone.

**Conclusions:**

Combination of doxorubicin and Apo2L/TRAIL is more effective in growth inhibition of PC3 xenografts *in vivo *than either agent alone and could present a novel treatment strategy against hormone-refractory prostate cancer. The intracellular mechanism by which doxorubicin enhances the effect of Apo2L/TRAIL on PC3 xenografts may be by reducing expression of c-FLIP.

## Background

Prostate cancer is a significant health problem among American men. This year about 230,110 men will be diagnosed with the disease and about 30,000 will likely die in this country alone [1]. Treatment options are limited and are associated with significant morbidity and mortality [2]. Localized cancer is treated with radical prostatectomy, brachy- or cryotherapy, and external beam radiation while cancer that has escaped the prostatic capsule is generally treated by androgen ablation. However, the eventual development of an androgen-independent phenotype leads to incurable disease, indicating the need for better treatment strategies. Experimental approaches include delivery of oncolytic viruses, immunomodulatory molecules, p53 and p21, enzymes that metabolize prodrugs and agents that can induce apoptosis [3]. One agent that has received considerable attention as a novel apoptosis-inducing agent is tumor necrosis factor-related apoptosis inducing ligand (TRAIL/Apo2L).

TRAIL is a type II membrane protein that can induce apoptosis by binding to death domain containing receptors DR4 and DR5 [4]. Unlike other death receptor ligands such as TNF and FasL, which cause septic shock and hepatotoxicity, respectively, TRAIL is tolerated well in mice and non-human primates [5-7]. TRAIL induces apoptosis in a variety of cell lines *in vitro *and *in vivo*. Apoptosis is initiated by binding to receptors DR4/DR5 (TRAIL-R1, 2), which is followed by assembly of the death inducing signaling complex and activation of caspase-8. Subsequent activation of caspases-3/7 (in a mitochondria-dependent or independent fashion) leads to execution of apoptosis [4].

Active caspase-8 tetramers are generated by cis- and transcatalytic cleavage from pro-caspase-8 homodimers [8]. These cleavage steps are inhibited by heterodimer formation of caspase-8 with c-FLIP. The apoptosis inhibitor c-FLIP is structurally similar to caspase-8 but lacks the cysteine residue essential for catalytic activity. A strong correlation between c-FLIP expression and malignant potential has been observed in carcinomas of the colon and liver as well as melanomas [9-11]. In addition, a high c-FLIP/caspase-8 ratio has been associated with resistance to death receptor-mediated apoptosis [11-13]. Thus downregulation of c-FLIP is a desirable strategy to enhance the apoptotic response to death receptor ligands.

We have previously shown that prostate cancer cells are relatively resistant to recombinant TRAIL but can be sensitized by pretreatment with the chemotherapeutic agent doxorubicin [7,14]. The enhanced susceptibility of prostate cancer cells was independent of androgen-phenotype or p53 status but correlated with doxorubicin-mediated downregulation of c-FLIP expression. In this study we extended those observations to an *in vivo *model using xenografts of PC3 prostate carcinoma cells.

Our results show that doxorubicin reduces c-FLIP expression in xenografts and that combination of doxorubicin with TRAIL is more effective in tumor growth inhibition than either agent alone.

## Methods

### Cells and reagents

PC3 cells were purchased from the ATCC and were maintained in RPMI1640 supplemented with 10% FBS at 37°C with 5% CO_2_. Apo2L/TRAIL was generously provided by Genentech Inc., San Francisco CA. KillerTRAIL was purchased from Alexis, San Diego, CA. Doxorubicin was obtained from the MUSC pharmacy. The CellTiter Aqueous One Solution Cell Proliferation Assay and DeadEndTUNEL kits were purchased from Promega, Madison WI. The c-FLIP antibody NF-6 was kindly provided by Dr. Marcus Peter, University of Chicago. The Dave-2 (c-FLIP) antibody and anti-actin were purchased from Alexis, San Diego, CA and from Sigma, St. Louis, MO, respectively. Supersignal DuraWest was obtained from Pierce Biotechnology Inc., Rockford, IL.

### MTS viability assay

Cells were seeded into 96-well plates at 1 × 10^4 ^cells/well, incubated overnight and subsequently treated with doxorubicin and/or Killer-TRAIL/Apo2L/TRAIL. The MTS [3-(4,5-dimethylthiazol-2-yl)-5-(3-carboxymethoxyphenyl)-2-(4-sulfophenyl)-2H-tetrazolium] reagent was added 24 hours after initiation of treatment and plates read at an absorbance of 490 nm one to two hours later using a Vmax kinetic microplate reader (Molecular Devices, Sunnyvale, CA). All treatments were performed in triplicate. Background absorbance was determined by incubating media with substrate alone and subtracting the values from wells containing cells. Percent cytotoxicity was calculated as follows: % cytotoxicity = 1 - [(OD of experimental/OD of control) × 100]. Experiments were repeated three times with similar results. There were no discrepancies between results obtained in the MTS assay and visual assessment of the cells prior to adding the MTS reagent.

### Animal experiments

Athymic male nude mice (3–4 weeks old) were purchased from Harlan, Indianapolis IN and were housed under pathogen-free conditions according to Medical University of South Carolina animal care guidelines. All animal experiments were reviewed and approved by the Institutional Animal Care and Use Committee at MUSC. PC3 cells (4 × 10^6^) were injected subcutaneously into the flanks of mice. Animals bearing tumors were randomly assigned to treatment groups (five or six mice per group) and treatment initated when xenografts reached volumes of about 100 mm^3^. Tumors were measured using digital calipers and volume calculated using the formula: Volume = Width^2 ^× Length × 0.52, where width represents the shorter dimension of the tumor. Treatments were administered as indicated using vehicle (PBS containing 0.1% BSA), doxorubicin (2–8 mg/kg), Apo2L/TRAIL (500 μg/animal), or a combination of 4 mg/kg doxorubicin followed by 500 μg Apo2L/TRAIL. Doxorubicin was administered systemically whereas Apo2L/TRAIL was given either intra-tumorally (Fig 4) or systemically (Fig 5). All treatments were given once. Mice were monitored daily for signs of adverse effects (listlessness and scruffy apparance). Treatments seemed to be well tolerated. The mean ± SEM was calculated for each data point. Differences between treatment groups were analyzed by the student t-test. Differences were considered significant when P < 0.05.

### Western blotting

Following doxorubicin treatment, mice were sacrificed, tissue removed and immediately frozen in liquid nitrogen. Protein was prepared in RIPA buffer containing freshly added mammalian protease inhibitor cocktail. Lysates were stored at -70°C and were centrifuged (20,000 × g) prior to performing protein assays on the supernatant. Protein (50 μg) was separated on 4–12% Bis/Tris NuPage gels in MES buffer and transferred to nitrocellulose for 60–90 minutes at 30 V. After transfer and blocking (5% milk), blots were probed with Dave-2 (1:500 in 0.5 % milk) or NF-6 (1:5 in TBS-Tween) overnight at room temperature. Following three washes with TBS-Tween, membranes were incubated with the anti-mouse (1:5,000) or anti-rat (1:15,000) HRP-conjugated secondary antibodies for 1 hour at room temperature in 5% milk in TBS-Tween. Membranes were washed three times in TBS-Tween followed by chemiluminescent detection of the secondary conjugates with DuraWest Supersignal. Membranes were reprobed with anti-actin (1:2000) to ensure equal loading.

### TUNEL staining

Tumors were excised and fixed in 10% formalin and processed by standard procedures. Sections were analysed for apoptotic cells using the DeadEND Tunel kit according to the manufacturers instructions. Stained slides were examined for TUNEL positive cells using a Zeiss Axiovert 200 microscope (20×). TUNEL positive cells from 4 fields/slide were counted by two investigators to calculate the mean ± SEM. P-values were calculated using the student's t-test.

## Results

### Comparison of KillerTRAIL and Apo2L/TRAIL *in vitro*

We have previously shown that the TRAIL apoptotic response in prostate cancer cells can be enhanced by doxorubicin [14,15]. These studies were conducted using KillerTRAIL, a commercially available form of the protein that is crosslinked for maximal activity and contains a histidine tag. Concerns about hepatotoxicity were raised when a polyhistidine tagged recombinant version of TRAIL induced apoptosis in human hepatocytes [16]. A subsequent study revealed that different recombinant versions of TRAIL vary widely in their biochemical properties and their potential to cause toxicity. TRAIL containing a histidine tag (Apo2L/TRAIL.His) contained less Zinc, had a less ordered conformation and was more heterogeneous than TRAIL without a tag (Apo2L/TRAIL.0) [17]. In contrast to Apo2L/TRAIL.His, Apo2L/TRAIL.0 was non-toxic to human hepatocytes. Thus non-tagged Apo2L/TRAIL.0 (referred to as Apo2L/TRAIL) is the preferable form to use in preclinical studies.

**Figure 1:**
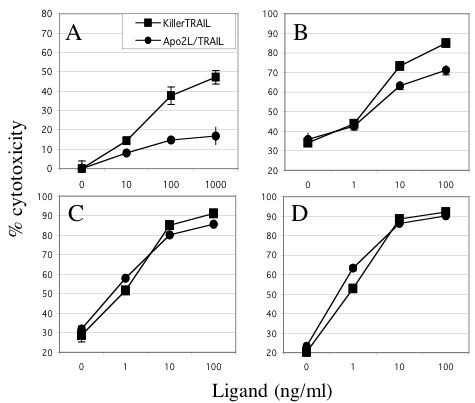
**Cytotoxic effects of KillerTRAIL and Apo2L/TRAIL *in vitro*. **PC3 cells were incubated with ligand at the indicated concentrations without (A), or with 0.25 μg/ml (B), 0.5 μg/ml (C) or 1 μg/ml (D) doxorubicin for 24 hours. Data shown are the mean ± SEM from a representative experiment. Similar results have been obtained three times.

Initially we compared the susceptibility of PC3 prostate carcinoma cells to KillerTRAIL and Apo2L/TRAIL in parallel assays *in vitro*. As shown in Figure 1A, at 1000 ng/ml KillerTRAIL resulted in about 50% cytotoxicity whereas less than 20% cytotoxicity was obtained with Apo2L/TRAIL. This difference may stem from the histidine tag of KillerTRAIL. In the presence of doxorubicin, KillerTRAIL and Apo2L/TRAIL were about equally effective (Fig 1B–D). Doxorubicin lowered the concentration requirement for TRAIL with near maximal killing achieved at 10 ng/ml ligand.

### Effect of doxorubicin on growth and c-FLIP expression *in vivo*

**Figure 2:**
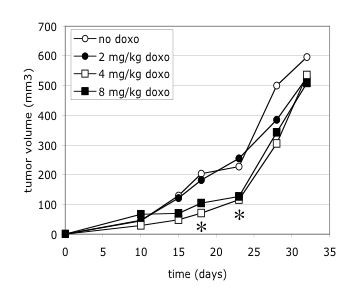
**The effect of doxorubicin on growth of PC3 xenografts. **Athymic nude mice were injected with PC3 cells (day 0) and treated with doxorubicin (i.p.) on day 10. Tumor size in animals treated with 4 or 8 mg/kg doxorubicin were significantly different from the untreated group on day 18 and 22, P < 0.05.

To determine an appropriate dose of doxorubicin for the *in vivo *experiments, mice bearing PC3 xenografts were injected with 2, 4 or 8 mg/kg doxorubicin and tumor volume was measured over time (Figure 2). A dose of 2 mg/kg did not affect tumor growth while higher dosages delayed tumor growth initially (p < 0.05 at days 18 and 22). However, no statictically significant differences were detected between untreated and doxorubicin treated groups at later time points.

**Figure 3:**
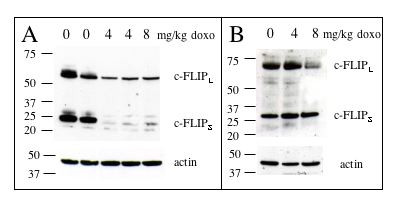
**Effect of doxorubicin on c-FLIP *in vivo ***Mice bearing PC3 xenografts were untreated or treated with 4 mg/kg or 8 mg/kg doxorubicin (i.p.). After 24 hours, protein was isolated from xenografts (A) or mouse heart (B) and probed for c-FLIP expression by western blotting. Actin was included as a loading control.

Previously, we have examined possible targets of doxorubicin that may be responsible for increasing the susceptibility of prostate cancer cells to TRAIL *in vitro*. These included TRAIL receptors, Bax, Bcl-2, Bcl-xl, and c-FLIP [15]. In PC3 cells, the only change observed in response to doxorubicin was a decrease in c-FLIP that correlated with onset and magnitude of caspase-8 activation and apoptosis. To investigate whether doxorubicin would have a similar effect on c-FLIP *in vivo*, protein from PC3 xenografts was isolated for western blotting 24 hours after systemic delivery of the drug. As shown in Figure 3A, we found that either 4 mg/kg or 8 mg/kg doxorubicin significantly reduced levels of c-FLIP in PC3 xenografts. The antibody used for the detection of c-FLIP (NF-6) is human-specific and thus detects only c-FLIP of PC3 origin. We also investigated the effect of doxorubicin on c-FLIP in the heart from the same mice. The heart was chosen because it expresses high levels of c-FLIP [18]. In addition, c-FLIP-deficient mice do not survive past day 10.5 of embryogenesis due to impaired heart development [19], indicating that c-FLIP plays a critical role in this organ. Protein isolated from the heart was analyzed with the rat monoclonal antibody Dave-2 that detects both mouse and human c-FLIP, although all c-FLIP detected should be exclusively of mouse origin, since PC3 cells are grown subcutaneously. Two major proteins, each migrating somewhat slower than the human c-FLIP isoforms, were detected with the Dave-2 antibody (Fig. 3B). The signal of the lower band (c-FLIP_S_) was not diminished following doxorubicin treatment, whereas the signal of the upper band (c-FLIP_L_) was slightly reduced following administration of 8 mg/kg doxorubicin. Since 4 mg/kg doxorubicin reduced c-FLIP in PC3 xenografts as effectively as 8 mg/kg without affecting endogenous mouse c-FLIP, this dose was chosen for combination therapy.

### Effectiveness of Apo2L/TRAIL doxorubicin combination therapy *in vivo*

**Figure 4:**
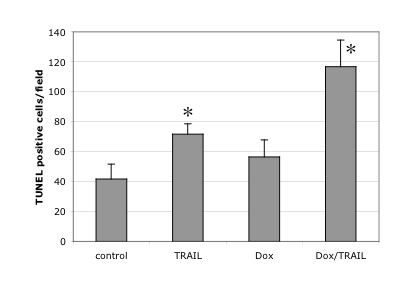
**TUNEL staining of PC3 xenografts **Mice bearing bilateral PC3 xenografts were untreated or treated with doxorubicin (i.p.) before receiving intratumoral injections of 500 μg Apo2L/TRAIL (left) or vehicle (right). After 24 hours, tumors were harvested, fixed, processed and analysed for TUNEL staining. Data shown are the mean ± SEM calculated from TUNEL positive cells counted in four fields.

To determine apoptosis *in vivo *following treatments, mice bearing bilateral PC3 xenografts were injected systemically with vehicle or 4 mg/kg doxorubicin, followed by intratumoral injection of vehicle (left tumor) or Apo2L/TRAIL (right tumor). Twenty-four hours after Apo2L/TRAIL injection, tumors were harvested and analysed for apoptosis by TUNEL staining (Fig. 4). There was no significant difference in TUNEL-positive cells in control and doxorubicin treated tumors. Treatment with Apo2L/TRAIL resulted in 70% more apoptotic cells than in vehicle treated tumors. However, combination treatment of doxorubicin and Apo2L/TRAIL yielded the most TUNEL positive cells (278% compared to the control) and was statistically different from all other treatment groups.

**Figure 5:**
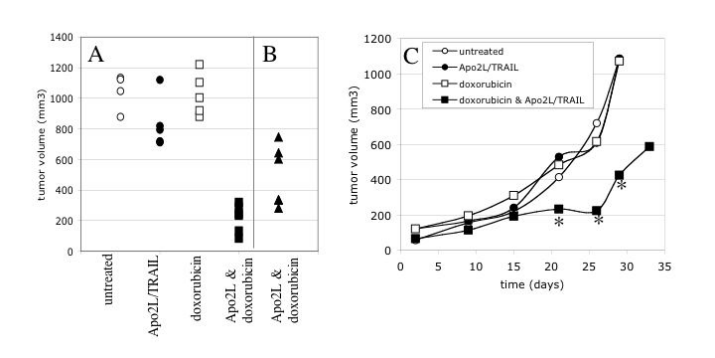
**Effect of combination therapy on the growth of PC3 xenografts **Mice bearing PC3 xenografts were untreated or treated with 4 mg/kg doxorubicin (i.p.), followed by either vehicle or 500 μg Apo2L/TRAIL (i.p.). Data shown represent tumor size twenty-six days after treatment was initiated (A). Tumor size in animals treated with combination therapy was monitored an additional week (33 days after treatment initiation) (B). Tumor growth over time (C) demonstrates that tumor growth is significantly different on and after day 21 (* indicates p < 0.05).

Next, we determined whether this pattern would be reflected in tumor growth of PC3 xenografts. Animals received systemic admininstration of doxorubicin followed by systemic injection of TRAIL after 24 hours, when c-FLIP levels were expected to have decreased. Tumors in animals injected with doxorubicin reached volumes of approximately 1000 mm^3 ^within 26 days after treatment initiation (1025 ± 138 mm^3^), which was not significantly different from the control group (1025 ± 118 mm^3^, p = 0.41). Tumors in animals treated with Apo2L/TRAIL were reduced compared to either untreated or doxorubicin treated groups (833.5 ± 150 mm^3^, p = 0.03). However, tumors that had been exposed to both doxorubicin and Apo2L/TRAIL were significantly smaller (224 ± 145 mm^3^, p < 0.001) and remained smaller at day 33 (490 ± 197 mm^3^) when the experiment was terminated. Analysis of tumor volume over time suggests that a single round of combination treatment delays growth until day 26 (Fig 5C). After tumors escape this delay they resume growth at rates similar to the other groups.

## Discussion

In this study we have shown that combination of Apo2L/TRAIL and doxorubicin is more effective in retarding tumor growth of PC3 prostate carcinoma xenografts than either agent alone. Doxorubicin is an anthracycline, which intercalates into DNA thereby activating DNA repair pathways and elevating levels of p53. PC3 cells are p53^-/-^, which may explain why doxorubicin as a single agent was relatively uneffective against tumor growth inhibition [20]. However doxorubicin was able to reduce expression of the anti-apoptotic protein c-FLIP *in vivo*. We have previously shown that sequential treatment of doxorubicin followed by TRAIL resulted in cell death *in vitro *[15]. Our data suggest that downregulation of c-FLIP is an important step *in vivo *that enhances TRAIL-induced apoptosis as evidenced by increased TUNEL staining in tumors treated with combination therapy. Other agents that reduce c-FLIP and increase death ligand mediated apoptosis include cycloheximide, 9-nitrocamptothecin, cisplatin, and the proteasome inhibitor PS-341 [21-24]. These agents affect multiple cellular responses suggesting that downregulation of c-FLIP may not be the sole factor by which death ligand-induced apoptosis is enhanced. However, selective reduction of c-FLIP using RNA interference or anti-sense technology is sufficient to sensitize human cell lines, including Du145 prostate carcinoma cells to death receptor ligands, indicating that c-FLIP is a major provider of resistance in this apoptotic pathway [25,26].

Tumors in mice that received Apo2L/TRAIL alone were about 20% smaller than those of the control group. Thus the growth inhibitory effect of Apo2L/TRAIL on PC3 xenografts *in vivo *was reflective of the results obtained *in vitro*. In contrast to single agent therapy, combination treatment with doxorubicin and TRAIL resulted in tumors that had 80% less volume than those in the control group. Tumors that received combination therapy continued to grow slowly, indicating that complete regression of PC3 xenografts was not achieved using a single treatment. In a recent study, PC3 xenografts were treated with irradiation followed by TRAIL, which resulted in complete growth inhibiton following three weekly rounds of therapy [27]. This indicates that multiple treatments may be neccessary to achieve a complete response.

We observed that a single administration of 4 mg/kg doxorubicin reduced cFLIP protein in PC3 xenografts but not mouse heart. One possibility is that cFLIP expression is differentially affected in normal versus malignant cells. If so, then doxorubicin would preferentially lower the apoptotic threshold in cancer cells and facilitate the selective elimination of these cells. Alternatively, the difference may be species specific. When grown in mice, human xenografts of various origins have successfully been treated by TRAIL in combination with other agents [5,24,27-29]. In contrast, combination of doxorubicin and TRAIL *in vitro *can induce cell death in normal human breast, mesothelial, or prostate epithelial cells, [14,30,31], which suggests that combination therapy may lack specificity when applied to humans. Could combination therapy in mice be non-toxic because endogenous mouse c-FLIP remains unaffected by chemotherapy? To avoid the possible complication of toxicity the effect of chemotherapy on c-FLIP expression in human patients should be carefully evaluated before considering systemic combination of chemotherapy and Apo2L/TRAIL.

## Conclusions

We found that combination of doxorubicin chemotherapy and Apo2L/TRAIL is more effective in tumor growth inhibition than either agent alone, indicating that this may represent a novel treatment strategy against prostate cancer. One of the mechanisms by which doxorubicin may enhance Apo2L/TRAIL apoptosis in PC3 xenografts is reduced expression of the anti-apoptotic protein c-FLIP. Future studies are needed to further investigate the effect of chemotherapy on c-FLIP in human patients and to optimize the treatment schedule to achieve complete tumor regression.

## Competing interests

The author(s) declare that they have no competing interests

## Authors' contribution

AZ carried out the animal experiments using doxorubicin and Apo2L/TRAIL and TUNEL staining. JM carried out the animal experiments using doxorubicin. CVJ carried out viability assays and western blot analysis, conceived of the study, participated in its design and coordination, and prepared the manuscript. All authors read and approved the final manuscript.

## Pre-publication history

The pre-publication history for this paper can be accessed here:
